# VDAC1 and the TSPO: Expression, Interactions, and Associated Functions in Health and Disease States

**DOI:** 10.3390/ijms20133348

**Published:** 2019-07-08

**Authors:** Varda Shoshan-Barmatz, Srinivas Pittala, Dario Mizrachi

**Affiliations:** 1 Department of Life Sciences and the National Institute for Biotechnology in the Negev, Ben-Gurion University of the Negev, Beer-Sheva 84105, Israel; 2 Department of Physiology and Developmental Biology, Brigham Young University, Provo, UT 84602, USA

**Keywords:** mitochondria, TSPO, VDAC1

## Abstract

The translocator protein (TSPO), located at the outer mitochondrial membrane (OMM), serves multiple functions and contributes to numerous processes, including cholesterol import, mitochondrial metabolism, apoptosis, cell proliferation, Ca^2+^ signaling, oxidative stress, and inflammation. TSPO forms a complex with the voltage-dependent anion channel (VDAC), a protein that mediates the flux of ions, including Ca^2+^, nucleotides, and metabolites across the OMM, controls metabolism and apoptosis and interacts with many proteins. This review focuses on the two OMM proteins TSPO and VDAC1, addressing their structural interaction and associated functions. TSPO appears to be involved in the generation of reactive oxygen species, proposed to represent the link between TSPO activation and VDAC, thus playing a role in apoptotic cell death. In addition, expression of the two proteins in healthy brains and diseased states is considered, as is the relationship between TSPO and VDAC1 expression. Both proteins are over-expressed in in brains from Alzheimer’s disease patients. Finally, TSPO expression levels were proposed as a biomarker of some neuropathological settings, while TSPO-interacting ligands have been considered as a potential basis for drug development.

## 1. Overview—The Translocator Protein, TSPO

The translocator protein (TSPO), formerly known as the peripheral-type benzodiazepine receptor (PBR) or the isoquinoline-binding protein, is an evolutionarily-conserved protein complex that binds benzodiazepines, such as RO5-4864, and isoquinoline carboxamide derivatives, such as PK11195 [1,2,3]. TSPO is a highly hydrophobic 18 kDa protein expressed both in prokaryotes (e.g., *Rhodobacter sphaeroides,* Q9RFC8) and eukaryotes (e.g., *Homo sapiens*: P30536), where it is predominantly located at the outer mitochondrial membrane (OMM), Recently, the 3D structure of TSPO in complex with (*R*)-PK11195 was obtained, revealing a complex structure characterized by the tight packing of its five α-helical transmembrane domains [4,5]. 

TSPO is involved in a wide variety of functions. These include cholesterol import and regulation of the mitochondrial membrane potential, as well as mitochondrial metabolism, apoptosis, cell proliferation, immunomodulation, inflammation, Ca^2+^ signaling, oxidative stress regulation, porphyrin transport, and heme synthesis [6,7,8,9,10,11,12]. Moreover, these effects are thought to be due to TSPO/VDAC interactions.

The influence of TSPO on gene expression and functional implications has been demonstrated by TSPO knockdown and by TSPO ligands, involving TSPO/VDAC interactions [13,14,15]. The function of TSPO in metabolism is reflected in studies demonstrating that in *Tspo* knockout mice, the absence of TSPO resulted in altered mitochondrial energy metabolism, together with lower oxygen consumption, membrane potential, and ATP levels [16]. TSPO also regulates mitochondrial energy homeostasis through the modulation of fatty acid oxidation in steroidogenic cells [17]. In addition, TSPO regulates autophagy by producing peri-mitochondrial domains of acute reactive oxygen species (ROS) that prevent the completion of protein ubiquitination by the ubiquitin ligase PARK2 [18]. It has been reported that TSPO deregulates mitochondrial Ca^2+^ signaling, resulting in increased levels of cytosolic Ca^2+^, leading to activation of the Ca^2+^-dependent NADPH oxidase (NOX), thereby increasing ROS levels [19]. Finally, TSPO inhibits mitochondrial autophagy (mitophagy), preventing essential ubiquitination of proteins [18]. 

## 2. The Voltage–Dependent Anion Channel VDAC1 

It has been proposed that TSPO forms a complex with the OMM protein voltage-dependent anion channel (VDAC) and the inner mitochondrial membrane (IMM) protein adenine nucleotide transporter (ANT) [20]. Of the three VDAC1 isoforms, VDAC1 is the major form expressed in most cells [21,22,23], and its interaction with TSPO has been studied.

**Figure 1:**
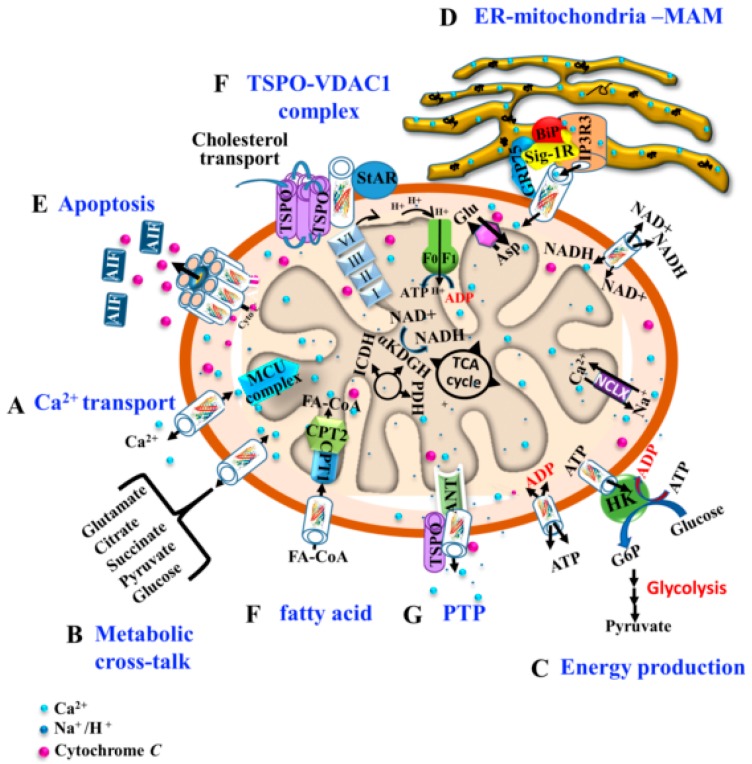
Schematic representation of VDAC1 as a multi-functional protein involved in Ca^2+^ and metabolite transport, energy production and the structural and functional association of mitochondria with the ER. The various functions of VDAC1 in cell and mitochondria functions are presented. These include: (A) Transporting Ca^2+^ across the OMM, thereby modulating Ca^2+^ signaling. In the IMM, Ca^2+^ uptake into the matrix is mediated by a Ca^2+^-selective transporter, the mitochondrial Ca^2+^ uniporter (MCU), regulated by a calcium-sensing accessory subunit (MCU1). Ca^2+^ efflux is mediated by NCLX, a Na^+^/Ca^2+^ exchanger. Ca^2+^ controls energy production via activation of PDH, ICDH, and α KGDH by intra-mitochondrial Ca^2+^, leading to enhanced activity of the citric acid cycle; (B) Control of metabolic cross-talk between the mitochondria and the rest of the cell, by transporting metabolites; (C) Mediating cellular energy production by transporting ATP/ADP and NADH and acyl-CoA from the cytosol to the IMS, and regulating glycolysis via the association with HK; (D) Involvement in structural and functional association with the ER, mediating Ca^2+^ transport from the ER to mitochondria. Key proteins, such as the inositol 3 phosphate receptor type 3 (IP_3_R3), the sigma1 receptor (Sig1R), the chaperone HSP70, and glucose-regulated protein 75 (GRP75) are presented; (E) Participation in apoptosis via its oligomerization to form a protein-conducting channel, allowing Cyto c release and cell death; and (F) Mediation of the transfer of fatty acid acyl-CoAs across the OMM to the IMS, where they are converted into acylcarnitine by CPT1a for further processing by β-oxidation. VDAC1 is involved in cholesterol transport as a constituent of a multi-protein complex, the transduceosome, containing Star, TSPO and VDAC1. (G) The permeability transition pore (PTP), composed of VDAC at the OMM, ANT at the IMM and Cyp D in the matrix, allows release of apoptogenic proteins.

Located in the OMM, VDAC1 serves as a mitochondrial gatekeeper, controlling a wide variety of mitochondrial functions (Figure 1) [23,24,25]. These include: (a) controlling the metabolic and energy cross-talk between mitochondria and the rest of the cell. VDAC1 transports solutes up to a molecular mass of 5 kDa into and out of mitochondria, including nucleotides (ATP/ADP and NADH/NAD), and metabolites (pyruvate, malate, succinate,). VDAC thus serves as a shuttle for respiratory chain substrates; (b) Ca^2+^ signaling by transporting Ca^2+^. VDAC1 is a Ca^2+^ channel that also transports Mg^2+^, Zn^2+^ and other ions. Once transported across the OMM via VDAC1, Ca^2+^ is transported across the IMM into the matrix by a Ca^2+^-selective transporter, the mitochondrial Ca^2+^ uniporter (MCU), which is regulated by a calcium-sensing accessory subunit (MCU1). In the matrix, Ca^2+^ regulates energy production via activation of pyruvate dehydrogenase (PDH), isocitrate dehydrogenase (ICDH) and α-ketoglutarate dehydrogenase (α KGDH), leading to enhanced activity of the citric acid cycle; (c) participation in mitochondrial-mediated apoptosis. Via homo-oligomerization to form a protein-conducting channel, VDAC1 allows cytochrome c (Cyto c) release and apoptotic cell death; (d) involvement, both structurally and functionally, in mitochondrial association with the ER, mediating Ca^2+^ transport from the ER to mitochondria; (e) involvement in lipid metabolism, mediating the transfer of fatty acid acyl-CoA across the OMM into the intermembrane space (IMS), where it is converted into acylcarnitine by CPT1a for further processing by β-oxidation; and (f) involvement in cholesterol transport as a constituent of a multi-protein complex, the transduceosome, comprising Star, TSPO, and VDAC1. 

Finally, VDAC1 is considered to be a hub protein, interacting with over 200 proteins [26] that regulate the integration of mitochondrial functions with other cellular activities. Thus, VDAC1 appears to be a junction for a variety of signals associated with cell survival and death mediated and regulated through association with various ligands and proteins.

## 3. TSPO Structure, Oligomeric State, Cholesterol Binding, and Interactions with VDAC1

### 3.1. TSPO Structure, Oligomeric State, and Cholesterol

Many high-resolution 3D structures of integral membrane proteins have proven to be fundamental for better understanding of many biological processes [27]. Nevertheless, considerations such as detergent choice, sequence homology to the target, and other parameters are crucial for exploiting the information obtained from crystallographic studies of a protein of interest [28].

**Figure 2:**
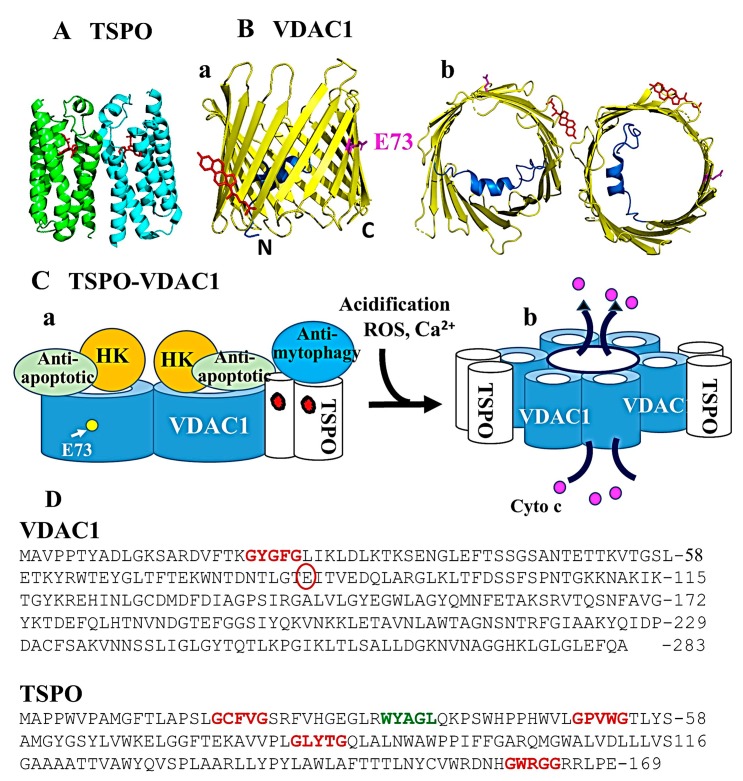
Structures of VDAC1, TSPO and their structural and functional complex (**A**) Crystal structure of dimeric *Bacillus cercus* TSPO (PDB ID: 4RYI) bound to PK11195. This structure is in good agreement with dimeric *Rhodobacter sphaeroides* TSPO (PDB ID:4UC3) and *Homo sapiens* TSPO structures solved with cholesterol analog PK11195 (PDB ID: 2MGY). Monomers of TSPO are in green and cyan, while PK11195 is in red [29]. (**B**) Crystal structure of human VDAC1 (PDB ID: 5XDN): (a) Monomeric VDAC1, (b) the dimeric form of human VDAC1. The N-terminal α-helix is in blue, cholesterol is in red, and E73 is in magenta. Cholesterol was manually docked for visual purposes [35]. (**C**) Proposed model for VDAC1-TSPO-associated functions: (a) In the OMM, VDAC1 and TSPO form dimers and associate with cholesterol. VDAC1 can be associated with hexokinase and anti-apoptotic proteins. TSPO is associated with anti-mitophagy partners to inhibit autophagy. Upon increased [Ca^2+^], acidification occurs, which in turn increases [ROS]. (b) Increased acidification, Ca^2+^ or ROS levels lead to VDAC1 oligomerization, concomitant with detachment of VDAC1 and TSPO-associated proteins. VDAC1 oligomers (likely hexamers) now create a large channel allowing the release of cytochrome c (Cyto c) from the IMS to the cytosol, activating apoptosis. TSPO is likely to stabilize the newly formed VDAC1 oligomer. (**D**) TSPO and VDAC1 sequences with the GXXXG motif labeled, and E73 in VDAC1 and the cholesterol-binding site in TSPO and the ATG8-binding motif (WYAGL, green) are also indicated.

There are currently 13 solved structures of TSPO (www.rcsb.org). Eleven are crystal structures and two are solution NMR structures. These structures were obtained from three different organisms, namely *Rhodobacter sphaeroides* (PDB ID: 4UC3 [29])*, Bacillus cercus* (PDB ID: 4RYJ [29])*,* and *Mus musculus* (PDB ID: 2N02 [30] and 2MGY [4]). The first structure was determined by Jaremko et al. [4] using NMR and mouse TSPO. In this initial case, the mouse TSPO structure was determined in the presence of 2% dodecylphosphocholine (DPC) and 2.9 mM (R)-PK11195, a ligand with nanomolar affinity in many species [31,32,33,34] (Figure 2A). 

Cholesterol has been shown to bind with nanomolar affinity to recombinant TSPO [32]. This interaction is due to the presence of a cholesterol recognition sequence ^147^ATVLNYYVWRDNS^159^ at the carboxylic terminus of TM5 [36,37]. The 3D structure of the TSPO-PK11195 complex revealed that the side chains of the essential amino acids Y152, Y153, and R156 [36,37] point towards the membrane environment. Site-directed mutagenesis of these residues inhibited binding of TSPO to cholesterol but not to PK11195 [36,37]. The location of residues essential for cholesterol binding at the outside of the TSPO structure, in combination with the known ability of cholesterol to dimerize, suggests that cholesterol binding can modulate TSPO oligomerization [29].

TSPO purified from *Rhodobacter sphaeroides (Rs)* was the first structure to present an oligomeric structure for the protein [29]. The RsTSPO structure was obtained from the lipidic cubic phase, a system that produces crystals of membrane proteins in more native-like oligomeric structures when compared to those obtained using detergents [38,39]. Crystal structures of TSPO (at resolutions of 1.8, 2.4, and 2.5 angstroms) from a mutant that mimics the human Ala147Thr polymorphism associated with psychiatric disorders and reduced cholesterol metabolism [40] was determined [29]. All three crystal structures show an identical interacting dimer and provide insight into the controversial physiological role of TSPO [29] and how the mutation affects cholesterol binding by perturbing the cholesterol binding site well characterized in the mouse TSPO-PK11195 structure [4]. TSPO also contains a binding domain for autophagy-related protein 8 (ATG8) [41] (W/YxxL/V/I) in its first cytosolic loop (Figure 2D).

Homology between eukaryotic and prokaryotic TSPO (~33%) is insufficient to extrapolate findings regarding oligomeric status suggested by the RsTSPO structure [42]. Nevertheless, an independent study of small homology domains can reveal conserved structural features between them. The evolutionary trace (ET) method [43] ranks amino acid residues in a protein sequence by their relative evolutionary importance, such that when an atomic structure is available for a protein, a structural map showing where top-ranked residues reside can also be generated. The ET annotation server (http://lichtargelab.org/software/ETserver) analysis of mouse TSPO (PDB 2N02) and RsTSPO (PDB 4UC1) assigned high relevance to approximately 10% of the homologous residues. These amino acids, found in both the prokaryotic and eukaryotic structures, are associated either with the cholesterol binding domain, as compared to region identified in the TSPO-PK11195 structure [4] or involved in dimer formation observed in the RsTSPO structure [29]. Thus, evolutionary studies point to a conserved function for TSPO.

The oligomeric state of TSPO in the OMM remains a matter of controversy. Blue native gels and electron microscopy [42] identified a dimer as the most likely organization of TSPO. This was confirmed in the crystal structure of RsTSPO in the lipidic cubic phase [29]. In addition to its atomic structure and possible aggregation states in the membrane, much remains a mystery in the case of TSPO. As mentioned above, TSPO interacts with a number of different proteins, although an ultra-structure of TSPO that correlates with its function in mitochondrial remains elusive.

VDAC1 is a channel protein comprising 19 transmembrane β-strands that form a β-barrel structure. A 26 residue-long N-terminal region usually lies inside the pore (Figure 2Ba), yet can also translocate outwards [21,44,45]. Mobility of this N-terminal region is important for channel gating and interaction with the anti-apoptotic proteins Bax, Bcl2, and Bcl-xL [23,24,46,47,48], as well as association with hexokinase (HK) [46,49] and VDAC1 dimerization [23]. VDAC1 has also been shown to present a cholesterol binding pocket (Ile123, Leu144, Tyr 146, Ala151, and Val171) [50]. As discussed above, TSPO binds cholesterol, with the crystal structure of TSPO-PK11195 (a cholesterol analog) having been determined [29]. 

VDAC1 is able to oligomerize, forming dimers, hexamers, and higher-order moieties (Figure 2Bb) [51,52,53,54,55,56,57,58,59,60,61]. Following the induction of apoptosis, monomeric or dimeric VDAC1 undergoes conformational changes to assemble into the higher oligomeric states that contain a large pore in the center of the oligomer that mediates Cyto c release and subsequent apoptosis [51,52,57,59,61,62,63]. 

### 3.2. TSPO and VDAC1 Interaction and Associated Functions

Another piece of evidence suggesting a conserved function for TSPO between prokaryotes and eukaryotes is the annotated database of protein-protein interactions (https://string-db.org/cgi/input.pl). The STRING database indicates that both RsTSPO and mammalian TSPOs interact with proteins related to ROS elimination, gene regulation and metabolism. RsTSPO binds proteins related to gene regulation, nutrients and ROS metabolism [64,65,66,67,68,69]. Human TSPO interacts with all three isoforms of VDAC. However, no model has been yet developed describing the interaction between VDAC1 and TSPO. The interaction of VDAC1 and TSPO in an environment devoid of native partners, in addition to interactions with the oligomeric states of VDAC1, may lead to the appearance of TSPO dimers so as to stabilize structures that create the larger VDAC1 pore (Figure 2C).

TSPO interacts with VDAC1 to contribute to the efficiency of the mitochondrial quality control machinery [18,26,70,71]. Via its interaction with VDAC1, TSPO inhibits mitochondrial autophagy downstream of the PINK1-PARK2 pathway, thus preventing essential ubiquitination of proteins [18].

The GxxxG motif has been implicated in alpha helical structures of membrane protein dimer formation [72]. The N-terminal domain of human VDAC1 (protein ID: P21796) contains one such GxxxG domain, 21-GYGFG. TSPO (protein ID: P30536) contains three GxxxG domains in its TM1, TM2, and TM3 transmembrane domains, 18-GCFVG, 50-GPVWG, 83-GLYTG. Additionally, the C-terminal end of TSPO also contains 160-GWRGG, although very little information was obtained from the NMR structure (PDB ID: 2MGY), where it appears disordered [29] (Figure 2D). Nevertheless, recent evidence indicates that disordered proteins can achieve structure upon binding to suitable partners [73]. If activation events lead both TSPO and VDAC1 to become free of native partners and change their oligomeric states, then it is possible that these proteins interact or establish a more stable partnership through their GxxxG motifs. The TSPO GxxxG motifs are, however, only present in eukaryotes. These motifs could, thus, differentiate structure-function relationships of mammalian TSPO beyond those seen in the ancestral prokaryote TSPO.

Finally, GxxxG motifs are broadly discussed as binding sites for ATP and cholesterol. On the other hand, they are accepted as relevant peptide dimerization/aggregation/membrane perturbation motifs that play roles in several pathological syndromes [7,74,75].

## 4. TSPO and VDAC1 Act in a Coordinated Manner

VDAC1 interacts with TSPO (see Section 3), with this interaction being potentially important for TSPO function [76], and may also affect TSPO ligand-binding characteristics [77,78]. Indeed, some TSPO activities involve VDAC1 [79,80]. It was also hypothesized [18] that the interaction of TSPO with VDAC1 contributes to the efficiency of mitochondrial quality control, regulating mitochondrial structure and function [18,81]. In addition, the accumulation of dysfunctional mitochondria, leading to mitophagy, is regulated by TSPO in a cholesterol-independent manner but is dependent on VDAC1 [18] (see Figure 2C).

TSPO involvement in the generation of ROS is proposed to link TSPO activation and VDAC1 and is considered to play role in the induction of mitochondrial-mediated apoptosis [1,2,82]. ROS appear to activate VDAC1 and facilitate VDAC1-mediated release of Cyto c from mitochondria to the cytosol [83,84]. It has been hypothesized that the close association of TSPO with VDAC1 [20,76] allows ROS generated via TSPO to act on VDAC1. Moreover, it has been suggested that TSPO grouping/polymerization around VDAC1 [2,78,85] may lead to high levels of TSPO-generated ROS in the vicinity of VDAC, leading to apoptosis activation [2,78,80]. In addition, an increase in cytosolic Ca^2+^ has been reported to lead to intracellular acidification [86] and VDAC1 oligomerization [87], and subsequently, to apoptosis.

Another proposed link between TSPO activity and VDAC1 is related to the regulation of cytosolic Ca^2+^. It has been proposed that TSPO controls intracellular Ca^2+^ dynamics, redox transients and cytotoxicity by increasing cytosolic Ca^2+^ levels via inhibition of mitochondrial Ca^2+^ uptake as a result of VDAC1 phosphorylation by protein kinase A (PKA) [19]. Such TSPO-dependent VDAC1 phosphorylation involves recruitment of PKA to the mitochondria, in complex with acyl-CoA-binding domain-containing protein 3 (ACBD3) [19]. Thus, elevated levels of intracellular Ca^2+^, acidification, and ROS involving TSPO regulate TSPO-VDAC1 interaction, the VDAC1 oligomeric state, autophagy and apoptosis [26,71,88] (Figure 2C). 

TSPO has also been suggested to activate the mitochondrial permeability transition pore (PTP) opening. The PTP represents a high-conductance, non-specific pore activated by ROS, Ca^2+^ overload, and other agents, leading to mitochondrial swelling and the release of Cyto c into the cytosol [89]. Initially, PTP was proposed to comprise VDAC1 in the OMM, ANT in the IMM, and cyclophilin D (CyD) in the matrix [90,91,92]. The physical interaction of ANT and VDAC is essential for PTP regulation [93]. However, in gene knockout experiments performed in mice, mitochondria from cells lacking some, but not all, ANT isoforms [94,95] or VDAC [96] still showed PTP formation. Recently, it was proposed that dimers of the ATP synthase complex could form the PTP [97]. Yet, several VDAC1-interacting molecules, such as HK [58], DIDS (4,4′-diisothiocyano-2,2′-stilbenedisulfonic acid) and G3139, potentiate PTP opening [98].

TSPO is proposed to regulate PTP opening, leading to ΔΨm transition and initiation of the mitochondrial apoptosis pathway [1,2]. The idea that TSPO is a component of the PTP is based on the finding that incubation of purified mitochondria with anti-TSPO antibodies delayed PTP opening and blocked the release of apoptosis-inducing factor (AIF) [99]. In addition, PTP opening was shown to be regulated by different TSPO ligands, such as the agonist Ro5-4864 and the antagonist PK11195, as well as the endogenous ligand PPIX [7,100,101]. However, the effects of TSPO ligands were proposed to be mediated by interaction with targets other than TSPO [102,103], including the mitochondrial ATP synthase [104]. Thus, the involvement of TSPO in the PTP structure and function requires additional studies. 

## 5. The Relationship between TSPO and VDAC1 Expression

**Figure 3:**
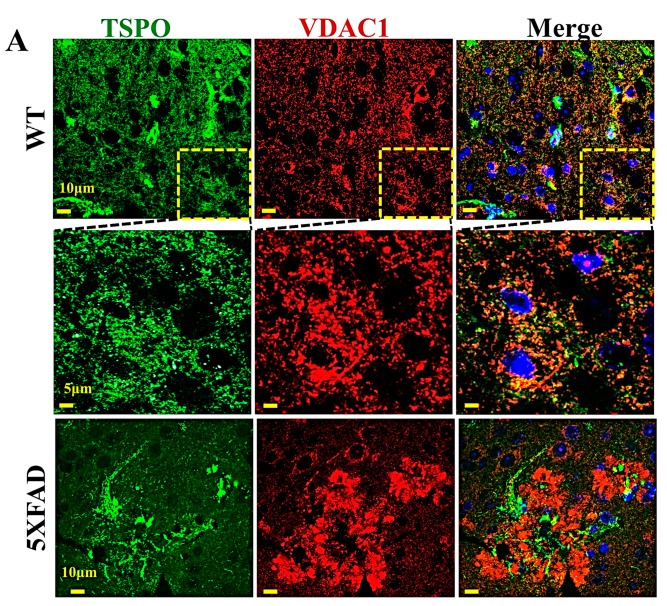

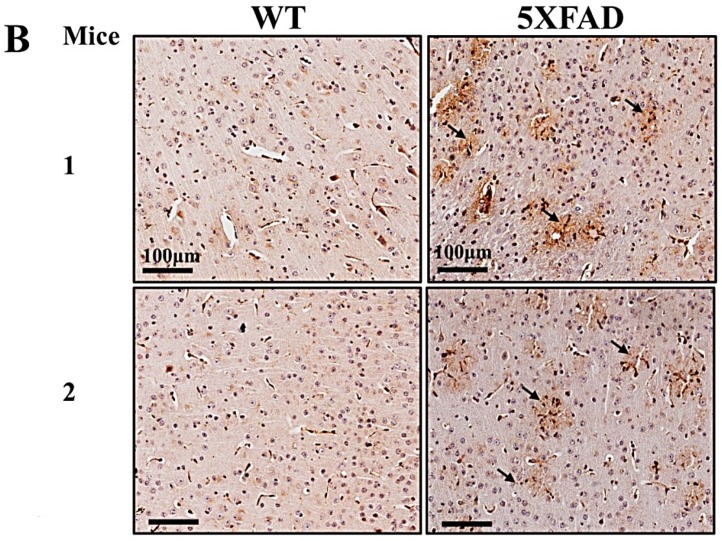
TSPO and VDAC1 are over-expressed in the brains of transgenic mice. (**A**) Cross-sections of brains from wild-type (WT) and 5XFAD transgenic mice, immunofluorescently stained for TSPO or VDAC1. Formalin-fixed and paraffin-embedded 5 μm-thick brain sections were deparaffinized, rehydrated, and subjected to antigen retrieval in 0.01 M citrate buffer (pH 6.0). For confocal fluorescence microscopic imaging of immuno-stained brain sections from WT and 5XFAD transgenic mice, the tissues were stained with anti-TSPO or anti-VDAC1 antibodies. Nuclei were stained by DAPI. Immunofluorescent staining were performed using mouse anti-VDAC1 (1:1000) and rabbit anti-TSPO (1:500) antibodies, followed by incubation (2 h, 25 °C) with secondary ant-rabbit Alexa-flur-488 or anti-mouse Alexa-Flu 555 (1:1000) antibodies. The cells were then stained with DAPI and viewed with an Olympus IX81 confocal microscope. (**B**) For immunohistochemistry, endogenous peroxidase activity was blocked by incubating the sections in 3% H_2_O_2_ for 15 min, after which the slides were washed and incubated overnight at 4 °C with primary rabbit anti-TSPO antibodies (1:200) and then for 2 h with anti-rabbit (1:500) secondary antibodies conjugated to horseradish peroxidase (HRP). Sections were washed and incubated with the HRP substrate, DAB. Images were collected at 20× magnification using a microscope (Leica DM2500). Non-specific control experiments were conducted using the same protocols but omitting incubation with primary antibodies. Arrows points to β plaques enriched with TSPO-expressing microglia.

TSPO is widely distributed in different tissues and is highly expressed in steroidogenic tissues, metastatic cancer, and upon inflammation (see Section 6). TSPO is also over-expressed in the brains of Alzheimer’s disease (AD) model mice (Figure 3). 

In our recent study, we demonstrated that down-regulation of VDAC1 in glioblastoma (GBM) also down-regulated TSPO expression [105]. Following GBM tumor treatment with si-RNA specifically targeting human VDAC1, not only were the levels of VDAC1 decreased but so were those of TSPO [105]. Such a decrease in TSPO expression levels suggests that TSPO was over-expressed in the tested tumors. As TSPO binds to, and acts, via VDAC1 [79,80,81], the observed decrease in TSPO levels may result from VDAC1 depletion or due to another unknown link. Interestingly, over-expression of TSPO inhibits VDAC1 expression, while silencing of TSPO increases VDAC1 expression in endothelial cells [79]. 

It has also been shown that in peripheral mononuclear cells (PBMCs) of bipolar disorder (BD) patients, both TSPO and VDAC mRNA and protein expression levels were highly increased, relative to their levels in healthy controls [106]. Moreover, the ratio of TSPO to VDAC was greater in BD than that in healthy controls [106]. In addition, it has been shown that the increase in mitochondrial ROS induced by an increase in the TSPO:VDAC1 ratio may activate protein kinase-Cε (PKCε) through the Raf-1-MEK1/2-ERK1/2 pathway, promoting the expression of TSPO [107]. Furthermore, increasing TSPO to VDAC1 ratios led to decreased mitochondrial ATP production, whereas ROS levels were increased, which subsequently inhibited PARK2-mediated ubiquitination, P62/SQTM1 recruitment and mitophagy. It is also shown that higher expression levels of TSPO and VDAC1 resulted in lower expression levels of Parkin and P62/SQSTM1, which may be related to a decrease in the activity of the mitophagic pathway [106]. 

TSPO and VDAC1 were also implicated in NLRP3 inflammasome formation [108]. The TSPO ligands Ro5-4864 and PK11195 effectively inhibited ATP-induced NLRP3 inflammasome activation via protection against mitochondrial perturbation. While increased VDAC1 levels led to activation of the NLRP3 inflammasome, down-regulation of VDAC levels in THP1 cells resulted in decreased caspase-1 activation and IL-1β secretion following inflammasome activation [109].

Taken together, these results indicate a relationship between TSPO and VDAC1 expression levels and several mitochondria-associated activities, such as ROS and ATP production, signaling and pathological conditions. The molecular mechanisms by which the interaction of TSPO and VDAC modulates these activities is not well-defined. 

## 6. TSPO and VDAC1 Expression in the Brain in Healthy and Disease States

While TSPO is expressed in many organs, the highest levels are seen in tissues containing steroid-synthesizing cells, such as adrenal glands, gonads and placenta [110,111]. TSPO is highly expressed in adrenal cortex and skin [112]. In brain, TSPO is predominantly expressed in glia, although low levels of TSPO were found in neurons [113,114]. Initially, TSPO expression in the brain was considered to be specific for activated microglia and infiltrating macrophages, thereby representing an inflammation biomarker [115,116]. However, it is now well established that reactive astrocytes also express TSPO [117,118]. Activated microglia proliferate, express TSPO, and release cytokines and other signal systems [119]. It has been shown that inactive glia express low levels of TSPO, while active cells show increased expression [115]. Accordingly, TSPO has been linked to glial and microglial cell activation. Such over-expression of TSPO in activated microglia and astrocytes in diseased brain is directly related to the degree of damage [113,120].

TSPO was found to be over-expressed in a variety of human diseases [116]. TSPO is up-regulated in various neuropathological conditions, including AD, Parkinson’s disease (PD) and multiple sclerosis (MS) [121]. Changes in TSPO expression have also been detected in psychiatric disorders [122]. In neurodegenerative disorders, such as AD, PD, MS, Huntington’s disease (HD), and amyotrophic lateral sclerosis (ALS), TSPO was found to be highly expressed at sites of injury and in microglia and astrocytes [107,118,123]. TSPO expression is significantly increased in activated microglial cells during brain inflammation in AD, PD, and other brain injuries [113,124,125]. It seems that TSPO offers a neuroprotective effect in these pathological conditions, as shown in AD mouse models [126] and MS [127]. 

Immunohistochemical staining of the brain from wild-type and an AD-like mouse model, APP/PS1dE9 transgenic mice, with anti-Aβ, anti-TSPO, and anti-Iba-1 antibodies (marker for microglia/macrophage), showed that plaque-associated Iba-1 was observed in cortex, hippocampus, cerebellum striatum, and thalamus, while plaque-associated TSPO was seen at all sites other than the thalamus [128].

Immunofluorescent staining of brain cortex sections for TSPO and VDAC1 they show punctuated and co-localized staining, as expected for two mitochondrial proteins (Figure 3A). Immunofluorescent staining of brain cortex in a 5XFAD mouse model that carries five familial AD (FAD) mutations [129], TSPO expression levels were highly expressed in microglia found in the Aβ plaque area (Figure 3A,B). In the brain cortex of 5XFAD transgenic mouse, VDAC1 is highly expressed in neurons around the Aβ plaque that are also enriched with TSPO-expressing microglia (Figure 3A).

Alongside the classical pathological hallmarks of AD, such as misfolded and aggregated proteins, neuro-inflammation is thought to be a major driver in progression of the disease [130,131]. Recently, TSPO was proposed as an indicator of brain neuro-inflammation [132]. Indeed, several TSPO ligands have been developed that allow for visualization of their binding for use as inflammation biomarkers [133,134,135]. 

The mechanism(s) of regulation of altered TSPO expression under various conditions is not clear. The broadly expressed transcription factors (TFs) acting on the TSPO promoter, such as specificity protein 1/specificity protein 3 (Sp1/Sp3), the v-ets erythroblastosis virus E26 oncogene homologue (Ets), and activator protein 1 (AP1), were proposed to regulate TSPO expression [136,137]. For example, the increased TSPO expression seen in microglia has been proposed to be attributed to TFs such as Sp1, Sp3, and GABP [138]. In addition, a transcriptional regulation of TSPO expression via a SINE B2-mediated natural anti-sense transcript (NAT) has been proposed [139]. However, the link between a specific pathological condition and activation of TSPO expression remain elusive.

## 7. TSPO and the TSPO-VDAC1 Complex as Targets for Neuro-Protective Agents

As TSPO is a multi-functional protein, expressed by different tissues and implicated in many pathological conditions [107,118,123], it has emerged as a candidate target for the development of compounds that modulate its activities. A variety of endogenous and synthetic ligands that interact with TSPO [140] were considered as a potential basis for drug development. These include the TSPO ligand XBD173, which exerts anxiolytic and anti-depressant effects [110], and 2 aryl-3-indoleacetamides (FGIN-1), which enhances mitochondrial steroidogenesis [141] and induces apoptosis [142]. In addition, porphyrines, phospholipase A2, and DBA, all endogenous TSPO ligands, have also been addressed. TSPO ligands have potential therapeutic applications, such as attenuation of neuro-protective effects [110], making them attractive agents for targeting neurological and psychiatric disorders [121].

In addition, due to the proposed function of TSPO in apoptosis, potentially via its interactions with VDAC1, interfering with the TSPO-VDAC1 interaction can offer a target for the development of drugs directed at neurodegenerative diseases [2,143]. 

In summary, these two outer mitochondrial membrane proteins, TSPO and VDAC1, are associated by direct interaction, and demonstrate functional and regulatory cross-talk. The link between the activation of TSPO and VDAC is proposed to play a role in cell proliferation and apoptotic cell death. In addition, there is a close relationship between TSPO and VDAC1 expression in healthy and disease states. Finally, as suggested for VDAC1 [25], TSPO expression levels were proposed as a biomarker for activated microglia and TSPO-interacting ligands have been considered as a potential basis for drug development. 

## Abbreviations

AD	Alzheimer’s disease
AIF	Apoptosis-inducing factor
ANT	Adenine nucleotide translocase
BD	Bipolar disorder
CyP	Cyclophilin
IMM	Inner mitochondrial membrane
OMM	Outer mitochondrial membrane
PTP	Permeability transition pore
ROS	Reactive oxygen species
TSPO	Transport protein
VDAC	Voltage-dependent anion channel
